# Supplementary material for: Secretory Nanoparticles of Neospora caninum Profilin-Fused with the Transmembrane Domain of GP64 from Silkworm Hemolymph
Source: Nanomaterials (Basel). 2019 Apr 10;9(4):593. doi: 10.3390/nano9040593 (PMC6523865; doi:10.3390/nano9040593)
Supplement: Supplementary file 1 [file nanomaterials-09-00593-s001.pdf]

Supplementary Materials

Secretory of nanoparticles *Neospora caninum* profilin fused with the transmembrane domain of GP64 from silkworm hemolymph

Hamizah Suhaimi[1], Rikito Hiramatsu[2], Jian Xu[3], Tatsuya Kato[1,2,3], Enoch Y. Park[1,2,3,*]

[1] Laboratory of Biotechnology, Department of Bioscience, Graduate School of Science and Technology, Shizuoka University, 836 Ohya, Suruga-ku, Shizuoka, 422-8529, Japan; noor.hamizah.binsuhaimi.16@shizuoka.ac.jp (H.S.); kato.tatsuya@shizuoka.ac.jp (T.K.); park.enoch@shizuoka.ac.jp (E.Y.P.)

[2] Laboratory of Biotechnology, Department of Applied Biological Chemistry, College of Agriculture, Graduate School of Integrated Science and Technology, Shizuoka University, 836 Ohya Suruga-ku, Shizuoka 422-8529, Japan; hiramatsu.rikito.17@shizuoka.ac.jp (R.H.); kato.tatsuya@shizuoka.ac.jp (T.K.); park.enoch@shizuoka.ac.jp (E.Y.P.)

[3] Laboratory of Biotechnology, Research Institute of Green Science and Technology, Shizuoka University, 836 Ohya Suruga-ku, Shizuoka 422-8529, Japan; xu.jian@shizuoka.ac.jp (J.X.); kato.tatsuya@shizuoka.ac.jp (T.K.); park.enoch@shizuoka.ac.jp (E.Y.P.)

[*] Correspondence: park.enoch@shizuoka.ac.jp ; Tel. & Fax: +81-54-238-4887

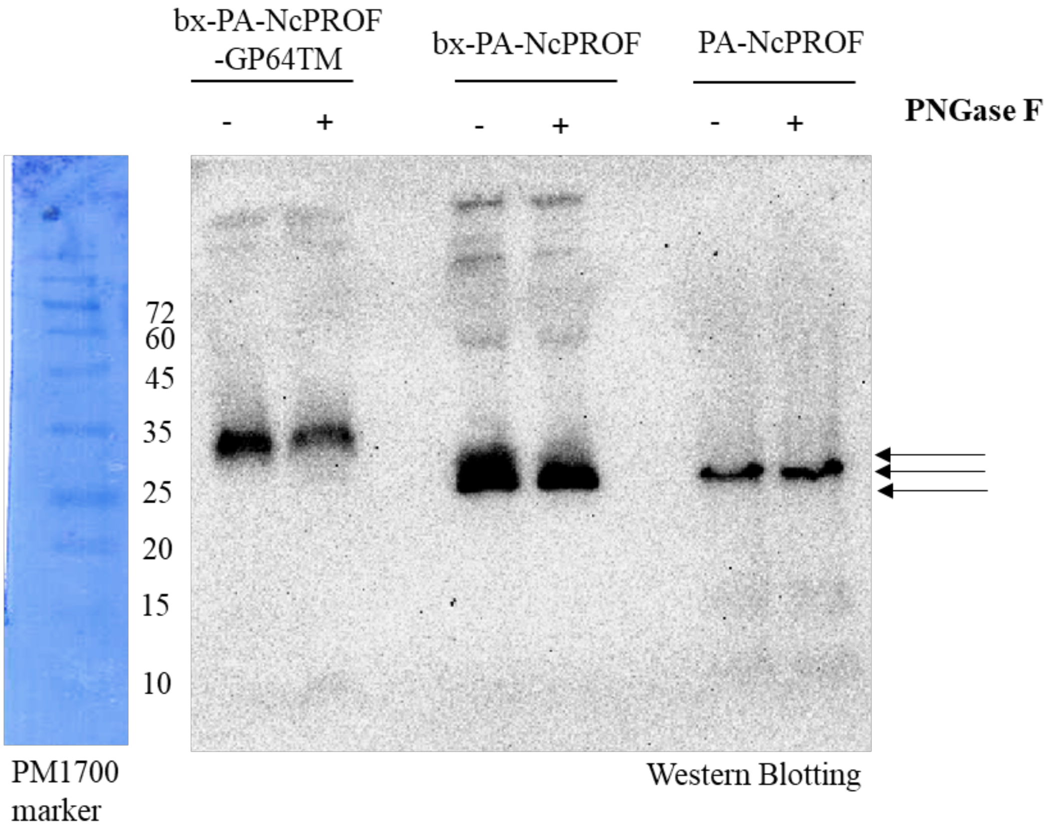

*SDS-PAGE Condition : 10% Gel Acrylamide, 150 V, 60 min, A: Constant

Figure S1. Analysis of *N*-Glycan attached to NcPROFs (bx-PA-NcPROF-GP64TM, bx-PA-NcPROF, PA-NcPROF) expressed in hemolymoh of silkworm. Western blotting with anti-PA tag antibody deglycosylated with PNGase F (incubation at 37°C for overnight) in denature condition. Upper, middle and lower arrows show the molecular weight of bx-PA-NcPROF-GP64TM, bx-PA-NcPROF and PA-NcPROF, respectively.

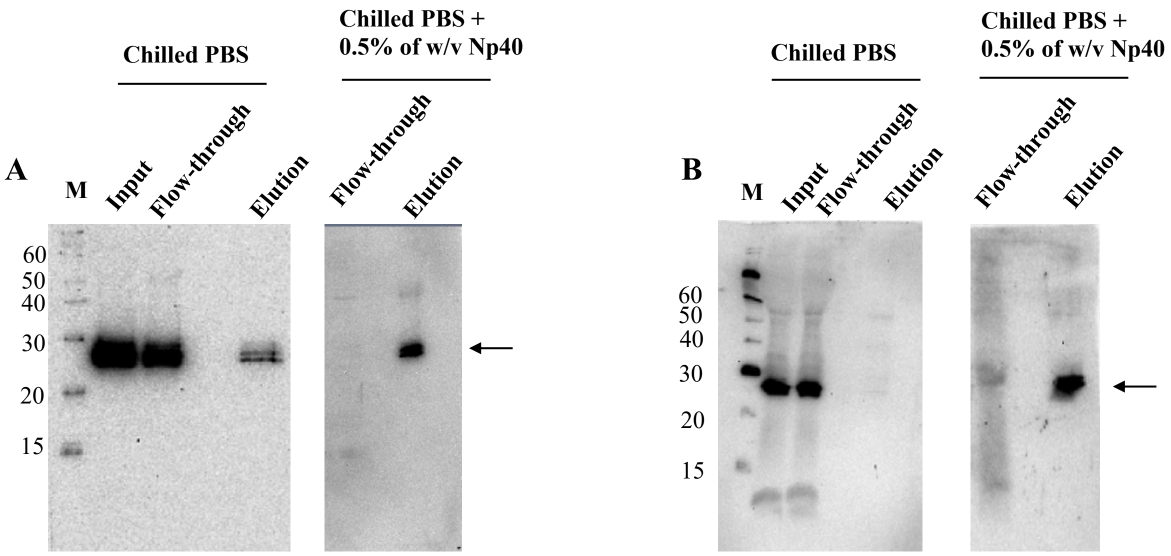

Figure S2. Western blots of purified bx-PA-NcPROF (**A**) and PA-NcPROF (**B**) from hemolymph with and without treatment with 0.5% of w/v Np40 using anti-PA tag affinity chromatography. M indicates molecular weight marker; arrows show the recombinant bx-PA-NcPROF and PA-NcPROF.

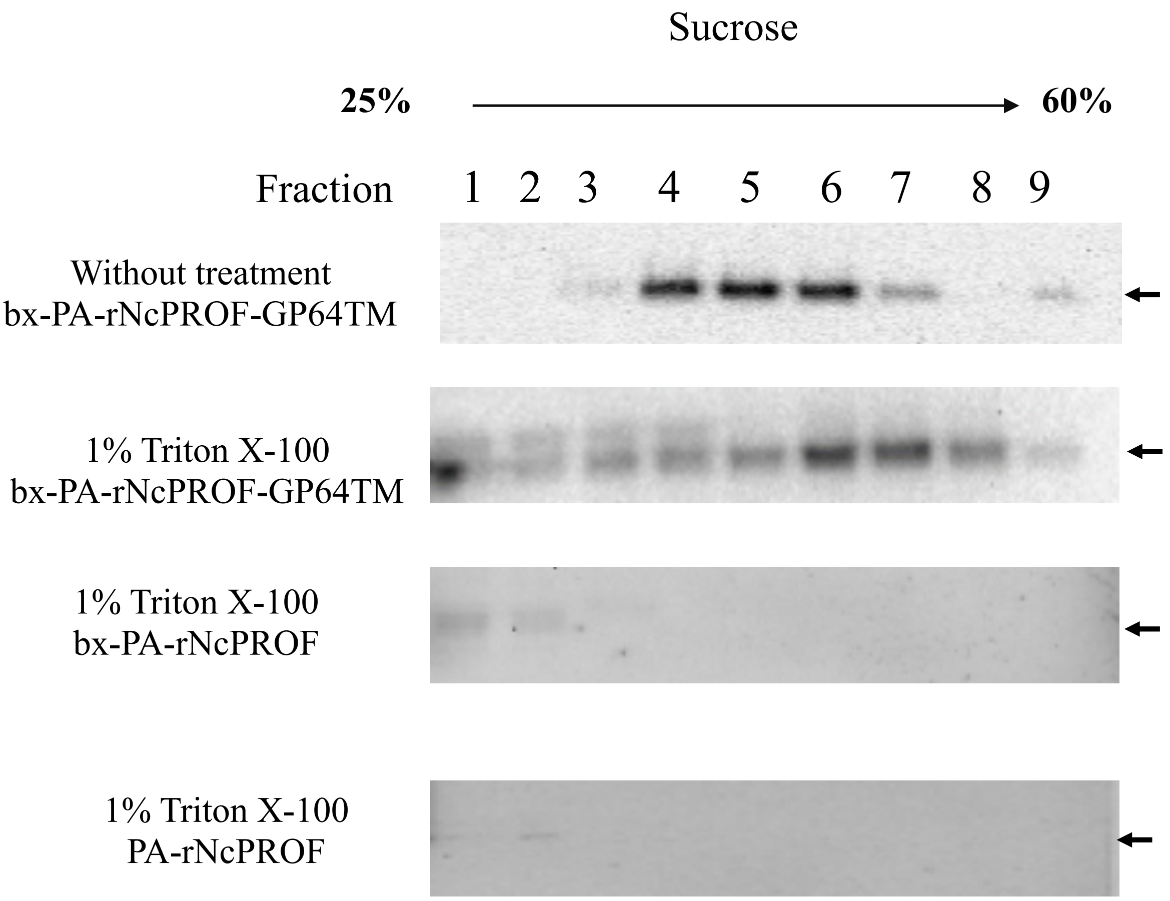

Figure S3. Western blotting analysis of incorporation level of bx-PA-NcPROF-GP64TM, bx-PA-NcPROF and PA-NcPROF with and without treatment of 1% Triton X-100.
